# Editorial: Chemoinformatics Approaches to Structure- and Ligand-Based Drug Design, Volume II

**DOI:** 10.3389/fphar.2022.945747

**Published:** 2022-06-29

**Authors:** Leonardo L. G. Ferreira, Adriano D. Andricopulo

**Affiliations:** Laboratory of Medicinal and Computational Chemistry, Center for Research and Innovation in Biodiversity and Drug Discovery, Physics Institute of Sao Carlos, University of Sao Paulo, Sao Carlos, Brazil

**Keywords:** drug design, machine learning, drug discovery, molecular docking, virtual screening, medicinal chemistry, QSAR

“*Chemoinformatics Approaches to Structure- and Ligand-Based Drug Design, Volume II*” follows the success of the first volume of this Research Topic (RT) (Ferreira and Andricopulo, 2018). The field has been more relevant than ever, especially in pandemic times, when Covid-19 hit the world and the scientific community was urged to come up with fast and cost-effective solutions (Robinson et al., 2022). Apart from the pandemic, chemoinformatics has been a core component in outstanding developments across different therapeutic areas and will continue to be a strategic innovation driver in the drug research and development (R&D) process (Chen et al., 2018; Ferreira and Andricopulo, 2019; Jiménez-Luna et al., 2021).

The second volume of this RT contains reviews and original research articles covering up-to-date research on machine learning (ML), multiparameter optimization (MPO), quantitative structure-activity relationships (QSAR), chemoinformatics servers, virtual screening, pharmacokinetics, among other equally relevant topics. More than 140 authors from all over the world contributed to the 20 articles that are part of this volume. Chemoinformatics investigations applied to different conditions such as Covid-19, cancer, Chagas disease, inflammation, pain, and immunological diseases are included. Additionally, novel approaches to pocket druggability analysis, multi-target drug discovery, artificial neural networks, multi-conformation molecular docking, molecular dynamics, and quantum studies are provided. Regarding target-based efforts, key aspects of intermolecular recognition are reported for a variety of proteins, including cruzain, G-protein coupled receptors (GPCR), phosphoglycerate mutase 1 (PGAM1), glutamate receptor, 5-lipoxygenase-activating protein (FLAP), Janus kinase 1 (JAK1), CC chemokine receptor 7 (CCR7), and cyclin-dependent kinase 2 (CDK2).

An MPO campaign combining computational and experimental approaches yielded a series of novel cruzain inhibitors (Pauli et al.). These compounds showed *in vitro* and *in vivo* trypanocidal activity along with low toxicity and suitable pharmacokinetics, contributing to the advance of Chagas disease drug discovery. Another study that integrated organic synthesis, biological evaluation, and molecular modeling (Oliveira et al.) resulted in the discovery of a series of carvacrol-derived sulfonamides with potent antioxidant, antinociceptive, and anti-edematogenic activities. Moreover, 3D-QSAR models (Wang et al.) were integrated with molecular docking and molecular dynamics to investigate anthraquinone-based PGAM1 inhibitors. Molecular modeling was also applied in combination with virtual screening and molecular docking to investigate novel inhibitors of JAK1 (Babu et al.), a critical enzyme for intracellular signal transduction and the development of numerous types of cancer. Given the importance of GPCRs in drug design, a review article covers recent discoveries on allosteric GPCR ligands and bitopic modulators (Egyed et al.). The role of the GPCR secondary site was examined in terms of its effects on properties such as binding affinity, selectivity, and kinetics. A further review article examines the currently available tools used to analyze molecular dynamics results (Baltrukevich and Podlewska).

Chemokines play a critical role in immunological signaling and, therefore, can be explored as drug targets in different diseases such as immunological and inflammatory conditions and cancer (Salem et al., 2021). A set of experimentally validated decoys were identified for CCR7 using a structure-based virtual screening approach (Proj et al.). In addition to the traditional single-target drug design paradigm, a new multitarget strategy is reported in this RT (Valdés-Jiménez et al.). A computational tool was developed to explore and identify druggable 3D arrangements across different proteins. This algorithm allows the comparison of quaternary structures and the evaluation of druggability from the 3D structural pattern. However, defining druggable and non-druggable protein cavities is neither a trivial nor an obvious task (Ehrt et al., 2019). Departing from the commonly used two-class classification models, a one-class approach to assess druggability (Aguti et al.) using a probabilistic kernel is communicated in this RT. The workflow proved to be feasible in removing or reducing biases in the classification of druggable pockets. Virtual screening has become an important tool in drug discovery as it allows a preliminary evaluation of large compound collections in short timelines and costs (Ferreira and Andricopulo, 2021). A novel virtual screening workflow (Venkatraman et al.) that can sample billions of compounds and supports parallel and cloud computing is reported. A collection of approximately 3.7 billion compounds against three Sars-CoV-2 proteins were used to evaluate the effectiveness of the new virtual screening pipeline. Another target-based study focuses on CDK2, which participates in the regulation of the cell cycle and is a critical player in cancer emergence. A series of aminopurine derivatives was designed as novel CDK2 inhibitors (Liang et al.) with high selectivity concerning other CDK isoforms. Anti-proliferative activity against triple-negative breast cancer cells (TNBC) was shown, which makes this series suitable starting points for optimization.

ML has been a hot topic in drug discovery, which is reflected in the number of articles on this theme published in this RT. Novel molecular targets for Covid-19 drug repositioning were identified (López-Cortés et al.) by a combination of artificial neural networks, single-cell RNA sequencing, and interactome analyses of the immunological system proteins. After a screen of more than 1,500 proteins, 25 putative molecular targets were identified. Interestingly, datasets containing more than 50,000 structurally diverse compounds with reported activity against several breast cancer cell lines were used to generate predictive models (He et al.). As a result, a web server was created to predict the activity of query compounds against breast cancer cell lines. Another online tool reported in this RT performs multi-conformational molecular docking (Wang et al.) on estrogen (ERα and Erβ) and androgen (AR) receptors. In addition, this interface runs 2D similarity searches against a database of known ERα, ERβ, and AR ligands. ML was also applied to identify the 2D features associated with the anti-inflammatory properties of FLAP inhibitors (Aliza Khan and Jabeen), which can assist the design of optimized anti-inflammatory agents. Furthermore, this RT brings to the readers an interesting analysis of the extrapolation limits of different regression methods (von Korff and Sander) applied to drug discovery along with an ML-based QSAR model (Brown et al.) for the estimation of molecular properties in drug design. In the field of deep learning, this article Research Topic features a report of a deep graph neural network (Shi et al.) to predict the interaction of small-molecule compounds with protein binding cavities. An additional important topic is drug resistance to antibiotics, which has emerged as a major health concern all over the world. ML has been applied to the field to identify novel chemical matter able to circumvent the main resistance mechanisms found in bacteria (Chowdhury et al., 2020). A review article examines recent machine learning studies (Jukič and Bren) applied to the identification of novel non-peptidic and peptidic antibacterial compounds and drug targets.

This RT encloses articles that cover a broad range of chemoinformatics applications to drug discovery and its many interfaces with the chemical and biological sciences. The knowledge shared through this RT could not be more relevant and timely. We hope that the findings, insights, and analyses reported herein contribute to the advance of drug discovery and, ultimately, to the promotion of human health.
